# The Effects of Silicone Enclosure Colour on the Function of Optical Sensors

**DOI:** 10.3390/biology11060932

**Published:** 2022-06-19

**Authors:** Garrett Frank, Shahbaz Askari, Katharina Raschdorf, Sadra Khosravi, Brian K. Kwon, Babak Shadgan

**Affiliations:** 1 International Collaboration on Repair Discoveries, 818 West 10th Avenue, Vancouver, BC V5Z 1M9, Canada; gfrank12@student.ubc.ca (G.F.); baz.askari@gmail.com (S.A.); katharin@student.ubc.ca (K.R.); sadrakh@outlook.com (S.K.); brian.kwon@ubc.ca (B.K.K.); 2 School of Biomedical Engineering (SBME), University of British Columbia, 2222 Health Sciences Mall, Vancouver, BC V6T 1Z3, Canada; 3 Department of Electrical and Computer Engineering (ECE), University of British Columbia, 2332 Main Mall, Vancouver, BC V6T 1Z4, Canada; 4 Department of Neuroscience, University of British Columbia, 2215 Westbrook Mall, Vancouver, BC V6T 1Z3, Canada; 5 Department of Orthopaedics, University of British Columbia, 2775 Laurel Street, Vancouver, BC V5Z 1M9, Canada

**Keywords:** implantable, biosensor, phantom, diagnostics, direct measurement, absorption, reflection, scattering

## Abstract

**Simple Summary:**

Implantable optical sensing is a rapidly growing field that allows for continuous monitoring of internal organs’ physiological states. Near-infrared spectroscopy is an optical sensing technology allowing for low-cost, non-invasive, high-sensitivity measurement of tissue oxygenation and haemodynamic parameters. The colour of an optical sensor’s enclosure affects the sensor’s sensitivity, function, and ability to detect tissue vital signs. This study compared the optical properties of coloured silicone materials and related these properties to the function of silicone enclosed implantable near-infrared spectroscopy sensors. We demonstrated that sensor enclosures highly reflective to red and near-infrared light facilitated light propagation to the photodetector and increased the ability to detect the effects of cardiac pulsation and respiratory rhythm on tissue haemodynamics. In contrast, highly absorptive sensor enclosures resulted in better detection and monitoring of tissue oxygenation.

**Abstract:**

The colour of the silicone enclosure of an implantable reflectance-based optical probe plays a critical role in sensor performance. Red-coloured probes that are highly reflective to near-infrared light have been found to increase photodetector power by a factor of 6 for wavelengths between 660 and 950 nm and triple the magnitude of measured cardiac pulsations compared to traditional black probes. The increase in photodetector power and cardiac pulsation magnitude is presumably due to increased spatial range resulting from a higher magnitude of superficial tissue scattering. Conversely, probes with highly absorbent colours such as black and blue result in more stable signals and are expected to have higher spatial resolution and depth of penetration.

## 1. Introduction

Optical biosensing is a rapidly growing field that enables real-time, low-cost, and high-sensitivity detection of a wide range of chemical materials and biological reactions [1]. Biosensors have experienced exponential growth in the fields of clinical diagnostics, food control, and environmental monitoring [2] due to having a high specificity with a small form factor [1]. Recent advancements in wearable biosensors have enabled the non-invasive monitoring of heart and respiration rate, blood and tissue oxygenation, temperature, and brain activity [3]. Wearable biosensing is an emerging field in athletics, capable of real-time monitoring of cardio-respiratory function, muscle fitness, body exertion, and injury recovery [4,5]. Implantable optical biosensing is also garnering interest as it provides more accurate and continuous physiologic monitoring of internal organs and deep tissues, thereby reducing the need for frequent clinical testing [6].

The physiologic state of internal tissues can be dissimilar to that of superficial tissues [7], rendering it difficult to detect and monitor the pathophysiology and function of deep organs with transcutaneous sensors. Implantable biosensors have been widely investigated for specific diagnostics and monitoring applications, such as monitoring blood glucose levels in type 2 diabetes and early diagnosis of organ dysfunctions. Implantable optical sensors, biosensors utilizing light as a transducer, are being used to monitor internal organ haemodynamics with key applications in monitoring brain activity [8], cancer detection, cardiac monitoring [9], and intraocular pressure monitoring in artificial corneas [10].

Near-infrared spectroscopy (NIRS) is an optical biosensing technique that utilizes the difference in absorbance of oxygenated and deoxygenated haemoglobin in the red and near-infrared spectrum to measure and monitor tissue oxygenation and haemodynamics [11]. NIRS is a low-cost, low-power solution that lends itself well to both wearable and implantable sensing applications. In addition to tissue oxygenation, NIRS can monitor heart and respiratory rates and rhythms by monitoring the haemodynamic effect of cardiac pulsation and respiration on tissue oxygenation level in real-time. While these physiologic parameters provide clinically significant information on the viability and haemodynamics of the tissue of interest, they are considered high-frequency noise and are often filtered out when assessing tissue oxygenation [12]. This introduces two competing objectives of using optical sensors: assessment of tissue viability and the evaluation of deep organ tissue oxygenation.

The design of implantable optical sensors faces technical challenges in biocompatibility [13], size requirements, and power economy [14]. Larger implantable devices require larger incisions, increasing the risk of patient discomfort, implantation and explantation complications, and tissue irritation. Device power consumption directly affects the required size of the power supply, electrical components, and heat sinks. Additionally, high-power sensors generate more heat, which may pose a thermal hazard. For biocompatibility, implantable sensors, particularly optical sensors, are often encased in implantable-grade silicone materials [15]. We hypothesize that the optical properties of the silicone enclosure affect the typical pathway of light through tissue, thereby controlling the trade-off between the ability to monitor tissue viability parameters (i.e., heart and respiratory rates) and the monitoring of organ haemodynamics and oxygenation parameters. Since tissue is a highly scattering low absorbance medium, a relatively large amount of superficial tissue scattering is expected, arising from both reflection at the tissue surface and scattering in superficial tissue layers [16,17]. This superficially scattered light can interact with the sensor enclosure, and their subsequent effects depend on the optical properties of the silicone enclosure. 

Transcutaneous optical sensors are typically encased in black enclosures to maximize the attenuation of ambient light and to ensure deep penetration of emitted light through absorbing superficially scattered light. Conversely, implantable optical sensors are shielded from ambient light and are positioned closer to the tissue of interest, thereby obviating the need for black enclosures. Pigments highly reflective to red and near-infrared wavelengths are expected to facilitate superficial tissue scattering, thereby increasing optical power reaching the detector through introducing additional potential pathways from the source to detector. Increased optical gain at the detector, in turn, allows for reduced light source power requirements facilitating miniaturized probe designs with improved power economy. Additionally, the suspected increase in the spatial range may increase the ability to detect physiologic signals, including cardiac pulsations and breathing cycles, leading to probes better suited to monitor tissue viability parameters. Conversely, the traditional choice of highly absorptive colours is expected to minimize superficial tissue scattering and mitigate noise sources such as sensor crosstalk to better monitor deeper layers. This study investigates the effect of pigment on silicone enclosures’ optical properties and their impact on the performance of implantable NIRS sensors in both benchtop and preclinical porcine studies. 

## 2. Materials and Methods

**Figure 1:**
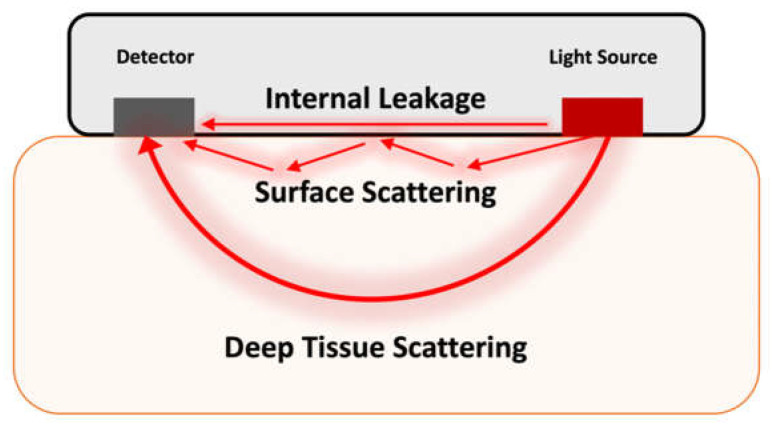
Expected pathways for light propagation from the light source to the detector.

The research was conducted in three phases: optical phantom preparation, optical analysis of phantoms using direct measurement, and benchtop studies using NIRS probes to determine how the optical properties of the silicone enclosure affect sensor signal and power requirements. Figure 1 displays a schematic of a typical implantable optical sensor and the possible pathways of light propagation from the light source to the detector.

### 2.1. Phantom Preparation

Optical phantoms were prepared using a transparent silicone elastomer (Sylgard 184, Dow, Midland, MI, USA). The silicone was pigmented with silicone dyes and titanium dioxide (TiO_2_) (PTR-630, Pantai Chemical USA, Alpharetta, GA, USA) as a scattering agent. Three silicone dyes were considered: red (Pantone Matching System (PMS) 186C, Silc Pig, Macungie, PA, USA), blue (PMS 2757C, Silc Pig, Macungie, PA, USA), and black (PMS Black, Silc Pig, Macungie, PA, USA). Dye concentration was 0.27% by mass to achieve phantoms sufficiently permissive for direct measurement. TiO_2_ was selected as a scattering agent due to its ease of implementation and widespread use in silicone phantom generation and commercial dye production [18]. TiO_2_ was added in three concentrations: 0, 0.018, and 0.036% by mass, concentrations found to predominantly result in single scattering events [19]. Table 1 lists the materials used for phantom preparation.

During preparation, the silicone dye and filtered TiO_2_ were added to the Sylgard 184 silicone. Following curing agent addition, silicone was thoroughly mixed using a custom mixer. The silicone was then transferred to the curing container, producing phantoms with a volume of 4 mL and a 30 mm diameter. Phantoms were then vacuum degassed to remove air bubbles and left to cure at room temperature. For each condition, three identical phantoms were produced from the same silicone base for a total of 27 optical phantoms.

### 2.2. Direct Optical Measurement of Silicone Phantoms

**Figure 2:**
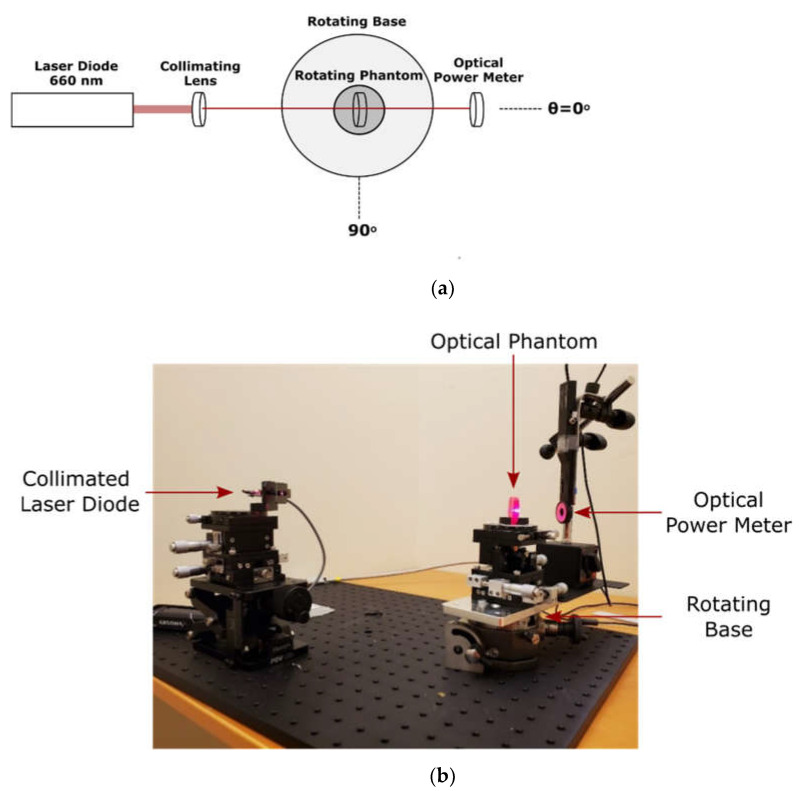
(**a**) Schematic of goniometric measurement technique; (**b**) the optical direct measurement apparatus.

The optical properties of produced phantoms were determined using goniometric techniques, shown in Figure 2. A collimated 660 nm laser diode (9687283011, Wal Front, Shenzhenshi, G.D., CH) was used as a light source. The optical phantom was fixed on the centre of a goniometer which was placed in the centre of a goniometric base. This apparatus allows for the independent rotation of both the optical phantom and the detector, an optical power meter (PM16-130, Thorlabs, Newton, NJ, USA). The attenuation coefficient was calculated using the Beer–Lambert equation, $I=I_{0}e^{\mu_{t}d}$, when both the phantom and detector were positioned at 0°. Additionally, the directionality of the scattering was assessed by measuring the optical power from 10° to 60° while keeping the phantom perpendicular to the source.

### 2.3. Benchtop Tissue Oxygenation Measurements

We have previously developed an implantable NIRS sensor to continually monitor spinal cord haemodynamics following acute spinal cord injury [20]. The sensor consisted of a customized multi-wavelength light-emitting diode (LED), and a photodetector mounted on a flexible printed circuit board and encased in an implantable medical-grade silicone rubber material. Optical power reaching the photodetector is linearly converted to an electrical current which in turn is converted to a voltage using a linear transimpedance amplifier. This raw photodetector value, measured in mV, is read by the controller and is proportional to the optical intensity at the photodetector. 

**Figure 3:**
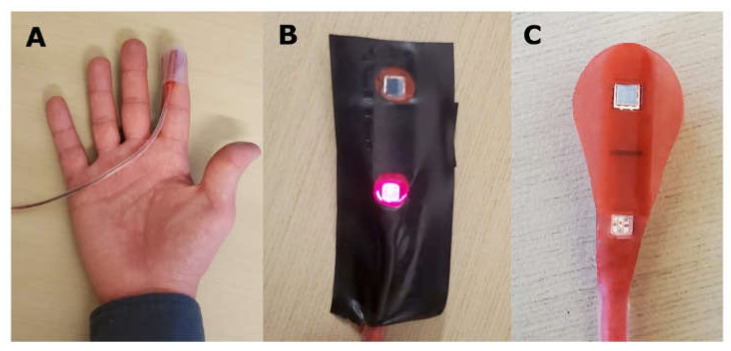
(**A**) Probe placement in benchtop studies; (**B**) black-taped probe; (**C**) red probe.

A similar probe was developed with a red silicone enclosure. The probe was placed on the index finger, and the light intensities of the sensor’s LED were adjusted so that the raw photodetector values were equal for each of the five emitted wavelengths: 660 nm, 730 nm, 800 nm, 850 nm, and 950 nm. Tissue oxygenation measurements were recorded *n* = 3 times on two individuals. Subsequently, to remove the red silicone–tissue interface, a single layer of black tape was applied to the probe. The probe was reapplied, the raw photodetector values were recorded, and tissue oxygenation measurements were repeated. The average amplitude and width of cardiac pulsations were manually assessed in each trial. Figure 3 displays the probe placement and tape application.

### 2.4. Animal Experiments

We are currently evaluating a customized NIRS spinal cord sensor for continuous monitoring of spinal cord oxygenation and haemodynamics in an animal model of acute spinal cord injury. We used this opportunity to evaluate and compare the function of NIRS sensors with red and blue silicone enclosures. All animal protocols and procedures performed were approved by the Animal Care Committee of the University of British Columbia (UBC) and were compliant with the policies of the Canadian Council of Animal Care and the U.S. Army Medical Research and Materiel Command (USAMRMC) Animal Care and Use Review Office (ACURO). The UBC Centre for Comparative Medicine established the anaesthesia/analgesia protocols. Two Yucatan minipigs weighing between 20 and 25 kg were used for these experiments. As described in previous articles, the animals were prepared for surgery, intubated, and anesthetized [21,22]. Briefly, animals were pre-medicated with intramuscular telazol (4–6 mg/kg), xylazine (1 mg/kg), and atropine (0.02–0.04 mg/kg). For anaesthesia induction, propofol (2 mg/kg) or isoflurane (2–3% in O_2_) were used before animals underwent endotracheal intubation. Propofol (8 mg/kg/h), fentanyl (12 µg/kg/h), ketamine (11 mg/kg/h), and midazolam (0.1–0.5 mg/kg/h) were used for anaesthesia maintenance through a continuous rate infusion (CRI). Anaesthetic levels were monitored via heart rate, respiratory rate, and blood pressure and adjusted as needed at the discretion of a veterinarian. The animals were mechanically ventilated for the duration of the surgery. Ventilator settings were adjusted throughout the surgeries as needed to maintain adequate tissue perfusion. 

**Figure 4:**
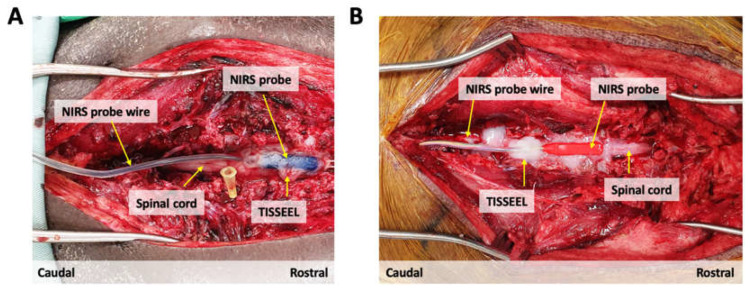
(**A**) Blue and (**B**) Red NIRS probe placement on the exposed dura of the porcine spinal cord during surgery.

In both animals, a dorsal laminectomy was performed between vertebral levels (A) Thoracic T6-Lumbar L1 and (B) Thoracic T1-Thoracic T5 to expose the dura membrane of the underlying spinal cord. The NIRS probes were subsequently tunnelled through the skin using a Hemovac surgical trocar and positioned on the exposed spinal cord dura at the level of (A) Thoracic T9 and (B) Thoracic T2/3. To secure placement and fixation of the NIRS probes over the underlying spinal cord dura and to prevent blood seepage underneath the sensors, the medical-grade fibrin sealant TISSEEL (Baxter, Mississauga, ON, CA) was applied around the edges of the sensor probes. After a one-hour baseline recording, we evaluated the NIRS sensor function by inducing an episode of hypoxia in both animals. This was achieved by pausing the ventilator until we reached an oxygen saturation of SpO_2_ 80% as measured by a peripherally attached pulse oximeter. At this point, ventilation was resumed, and the animals were allowed to recover. Oxygenated haemoglobin (O_2_Hb) signals were collected at a sampling frequency of 40 Hz. Figure 4 displays sensor fixation.

## 3. Results

For direct optical measurement of silicone phantoms, measured light intensity is expressed as a percentage of total emitted light. For tissue oxygenation measurements, both benchtop and animal comparison, the O_2_Hb signals were scaled such that baseline averages were equal. Cardiac pulsation amplitude is expressed in relative, arbitrary (ab) units.

### 3.1. Direct Measurement

**Figure 5:**
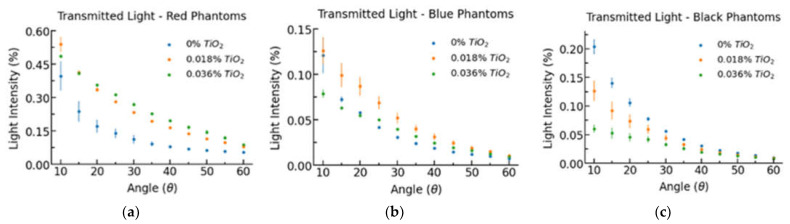
Transmitted light per scattering angle for (**a**) red phantoms; (**b**) blue phantoms; (**c**) black phantoms.

Figure 5 displays the measured optical power at each measured scattering angle, and Table 2 lists the average attenuation coefficient for each phantom based on colour and TiO_2_ concentration by mass. The attenuation coefficient for red phantoms is considerably smaller than for black and blue phantoms. The attenuation coefficient for red phantoms is approximately linear to TiO_2_ concentration. In contrast, blue and black phantoms do not share this linear relationship, having a larger than expected increase in attenuation from 0.018% to 0.036% TiO_2_. 

As shown in Figure 5, increased TiO_2_ concentration increases the light intensity at high scattering angles relative to low scattering angles, implying that TiO_2_ has a low asymmetry parameter defined as the cosine of the average scattering angle. In red phantoms, increased TiO_2_ concentration increased light intensity for every scattering angle between 10° and 60°. In black phantoms, increased TiO_2_ concentrations decreased light intensity at each scattering angle. Despite having a larger attenuation coefficient, black 0% TiO_2_ phantoms had a higher light transmission at every non-zero scattering angle compared to blue 0% TiO_2_ phantoms. These results imply that the black dye utilized is more strongly scattering than the blue dye.

### 3.2. Benchtop Tissue Oxygenation Measurement

The red sensor alone was used first to measure NIRS light transmission to the photodetector. The LED emission was calibrated to harmonize the raw photodetector values, resulting in a value of 3800 mV across all wavelengths. The application of black tape to the red probe replaced the highly reflective red silicone–tissue interface with a black surface, thereby mimicking a black sensor enclosure. This resulted in a 6-fold decrease in raw photodetector values, corresponding to a 6-fold reduction in optical power reaching the detector, as shown in Table 3. Additionally, 650 nm light appears to be most affected by the tape application; the one wavelength utilized within the red spectrum.

**Figure 6:**
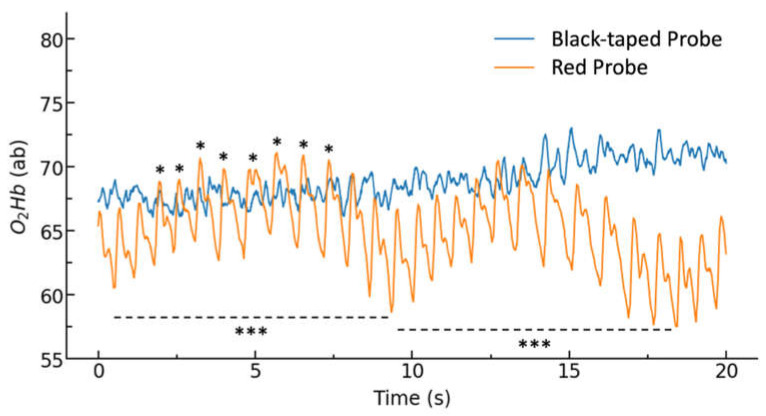
O_2_Hb baseline measurements for un-taped (red probe surface) and taped probes (black probe surface). * Cardiac pulsation, *** Respiratory cycle during controlled respiration.

Figure 6 displays the tissue oxygenation measurements for the red probe before and following the application of black tape. Table 4 lists the average amplitude and peak width of measured cardiac pulsations for each trial, as manually calculated over 10 pulsations starting 20 s after recording. In P01 trial 3, pulsation parameters were calculated after 10 s of recording due to motion artifacts present in the 20 s region. 

Tape application resulted in an approximately 3-fold reduction in cardiac pulsation amplitude and a loss of the ability to detect respiratory cycles. The relatively large trial-to-trial variability is due to small variations in sensor placement paired with a high sensitivity probe.

### 3.3. Animal Experiments

**Figure 7:**
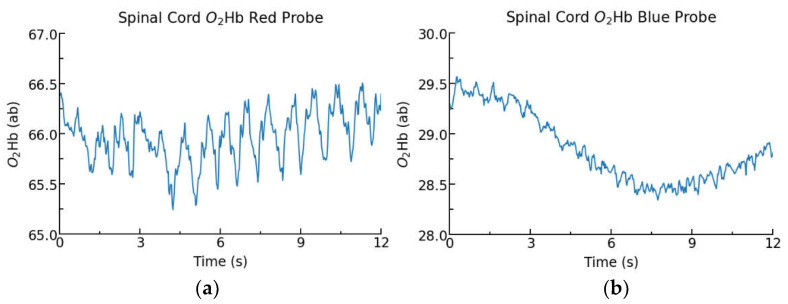
Spinal cord O_2_Hb measurements from a red (**a**) and blue (**b**) coloured probe in two separate Yucatan minipig experiments.

Figure 7 displays O_2_Hb measurements collected from blue and red probes placed on the spinal cord of two Yucatan minipigs in two separate experiments. The red probe provides more regular oscillations, which are cardiac pulsations, whereas the blue probe shows the O_2_Hb signal with lower oscillations but a clear changing pattern of tissue oxygenation. These results are consistent with the benchtop studies which compare the reflective red probe to a black enclosure, another highly absorbent, minimally reflective colour.

Table 5 displays the LED currents for the blue and red probes following wavelengths’ power adjustments. In both experiments, the sensor was calibrated by tuning LED powers to achieve a similar magnitude of light intensity at the detector for each wavelength. This calibration process was conducted to achieve the optimal signal performance for each probe within their hardware limitations. To achieve a similar photodetector power, the blue probe required 2.4-fold increased total LED current compared to the red probe. Lower wavelengths, closer to the red spectrum, required larger corrections for the blue probes. These results are consistent with the benchtop study.

## 4. Discussion

We have shown that NIRS sensors with red silicone enclosures facilitate increased photodetector power and improved ability to monitor tissue viability parameters (cardiac pulsations and respiratory effects) compared to black and blue enclosures, likely through supporting superficial tissue scattering. Conversely, highly absorbent blue and black silicone appear to favour deep tissue scattering, presumably producing signals with a higher spatial resolution, making them suitable for tissue haemodynamics and oxygenation monitoring. Within our benchtop and in vivo porcine spinal cord tissue oxygenation studies, we have shown that red-coloured probes facilitate the detection of physiologic signals, including cardiac pulsations and respiratory cycles. Furthermore, we have developed a simple approach to evaluate the optical properties of silicone-based optical phantoms to assist the design of the silicone enclosure based on sensor application. This preliminary investigation has shown that enclosure pigment is a critical design choice for implantable optical sensors and that the standard of highly absorbent enclosures are not optimal for all sensing applications. The evolution of this research, particularly in relating measurable optical properties of sensor enclosures to NIRS-specific parameters such as depth of penetration, can produce a toolkit, allowing developers a means of rapidly evaluating and selecting the optimal pigment for each unique project.

Within the optical phantoms, TiO_2_ was evaluated for two reasons. The addition of a scattering agent allows for fine control of silicone colour and as a potential means of increasing light attenuation within the enclosure. Since TiO_2_ tends to settle down prior to phantom curing, a lower concentration can be expected along the top surface [18,23], thus TiO_2_ addition should have a minimal effect on the reflectivity of the sensor–tissue boundary. For TiO_2_ concentrations that result in predominantly single scattering events, attenuation is expected to have a linear relationship with TiO_2_ concentration [19]. Produced red phantoms appear to follow this relationship, whereas blue and black do not. This suggests that different dye pigments can have considerably different scattering profiles, making certain pigments, including blue and black, more prone to multiple scattering. In order for multiple scattering to be negligible, $\mu_{t}d\ll 1$, where d is sample thickness [24]. This relationship only holds true for red 0% TiO_2_ phantoms, suggesting the potential for multiple scattering in other phantoms.

It is important to note that the relatively low attenuation of red silicone may increase the risk of internal optical leakage, or crosstalk, which can be expected to reduce the sensitivity of optical sensors for monitoring tissue oxygenation. The addition of TiO_2_ increased the attenuation coefficient of red silicone by a factor of 4.4, suggesting that the addition of a scattering agent into the optical probe enclosure may be an effective method for reducing internal optical leakage. However, manufacturing optical probes with TiO_2_ can be challenging as it readily precipitates out of solution prior to curing [25], which can have implications on both sensor-to-sensor variability and biocompatibility if precipitates form on the borders of the enclosure.

In red phantoms, increased TiO_2_ concentration increased light intensity for every scattering angle between 10° and 60°. This suggests that the addition of scattering agents to red probe enclosures may be effective in reducing sensor crosstalk by increasing the attenuation coefficient and can act to further increase spatial range by scattering light from the silicone into the tissue. In black phantoms, increased TiO_2_ concentrations decreased light intensity at each scattering angle, suggesting the presence of multiple scattering, thereby causing a higher-than-expected light attenuation through increased photon mean free path [26]. A similar effect can be seen with blue 0.036% TiO_2_ phantoms that exhibited lower intensity at every scattering angle than at 0.018% TiO_2_ suggesting the dominance of multiple scattering. Additionally, it is interesting to note that despite exhibiting a larger attenuation coefficient, black 0% TiO_2_ phantoms showed higher intensity at every scattering angle than blue 0% TiO_2_ phantoms. This finding suggests that the black dye used within this experiment is more strongly scattering and less absorbent than the blue dye, but these conclusions must be confirmed via additional testing such as the well-established single integrating sphere approach [27].

In the benchtop tissue oxygenation studies, the application of black tape to the red probe resulted in a 6-fold decrease in raw photodetector values. These results support the hypothesis that red-coloured probes facilitate increased light propagation to the photodetector, thereby reducing probe power requirements and enabling miniaturized designs. 660 nm light appeared to have the largest decrease in raw photodetector power following tape application. This result is intuitive as 660 nm light is within the red spectrum, and thus should have a larger magnitude of reflectance and backscattering from the red-coloured probe compared to near-infrared wavelengths. The large increase in photodetector power with red enclosures is significant as it aligns with the increased importance of power economy, size, and thermal stability for miniaturized implantable devices [28]. Additionally, the increased transmission of light to detector may improve sensor performance for light wavelengths > 900 nm wherein tissue absorbance substantially increases due to the increased absorbance of water in this range [29].

As expected, the application of black tape resulted in an approximately 3-fold reduction in cardiac pulsation amplitude. This is thought to be due to the loss of superficial scattering, decreasing the spatial range of the sensor to favour only deep scattering. As such, there is expected to be a lower probability that the light hitting the detector has passed through a tissue strongly affected by alternating current (A.C.) signals, including cardiac pulsations. Additionally, the tape application largely prevented the ability to detect the lower frequency respiratory patterns. These results are consistent with the observation that direct current (D.C.) signals, including the effects of non-pulsatile arterial blood, veinous blood, and tissue, increase with respect to A.C. components with increasing light penetration depth [30].

Similar results were observed within the porcine spinal cord oxygenation study, comparing the performance of blue and red-coloured probes. The highly absorbent blue probe required a 2.4-fold increase in total LED current to result in similar optical power at the photodetector. Despite the increased power consumption of the blue probe, cardiac pulsations were not as clearly visible compared to data collected from the red probe, as shown in Figure 7. However, data captured from the blue probe was able to detect low-magnitude patterns of changing O_2_Hb. It is important to note that the physiologic signals from cardiac and respiratory effects can be readily filtered from NIRS data [31], however red probes are expected to produce a higher noise signal due to their lower absorbance and higher reflectivity than highly absorbent probe enclosures. 

**Figure 8:**
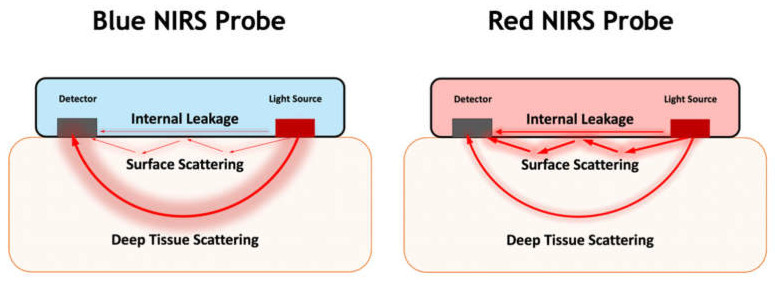
The expected effect of silicone colour of the NIRS sensor’s probe on deep tissue scattering, surface scattering and internal leakage of the NIRS sensor.

Our results suggest that highly attenuating enclosure colours, such as blue and black, provide high spatial resolution signals specific to deep tissue layers. Consequently, these enclosures are well suited to continuous monitoring of tissue haemodynamics and oxygenation, as in our NIRS spinal cord sensor. Highly reflective enclosure colours, such as red, have reduced power requirements with an increased ability to assess tissue viability parameters. These probe colours are expected to be well suited to applications such as monitoring the viability of tissue and organ transplants. Reflective probe enclosures may be desirable in the recent research trend in developing NIRS based sensors to monitor organ transplant perfusion and photoplethysmography (PPG) sensors [32,33,34]. Figure 8 demonstrates the predicted differences in light propagation within a blue and red NIRS probe. Our findings may be generalized to transcutaneous sensors, provided that the coloured sensing area is surrounded by a highly attenuating enclosure to mitigate the effects of ambient lighting. We expect white-coloured probe enclosures, which are highly reflective to visible and near-infrared light, to have a similar effect on sensor function as red probes. However, since white pigments are more highly scattering at a wider wavelength spectrum than red pigments, we hypothesize that white probes would be more attenuating, thereby reducing sources of noise such as internal leakage. 

A limitation of this study is the small sample size considered. Evaluation of a larger sample size should be performed to confirm our findings. Furthermore, additional testing should be conducted to confirm the theory that reflective sensor enclosures increase spatial range through facilitating superficial tissue scattering, whereas attenuating enclosures (blue and black) produce a high spatial resolution signal specific to deep tissues. One approach to verify this is an optical fibre punch through method to measure penetration depth in a tissue simulating optical phantom [35]. If red enclosures increase the magnitude of superficial tissue scattering, increased light intensity should be measurable in superficial layers. Furthermore, embedding a maximally absorbent, minimally reflective material at the maximal penetration depth in the phantom should resultantly cause a larger decrease in photodetector power for blue and black enclosures than for reflective enclosures.

## 5. Conclusions

The colour of the silicone enclosure for an implantable optical sensor plays a critical role in sensor performance and behaviour. Our results suggest that maximally absorbent and minimally reflective pigments, including blue and black, inhibit superficial scattering, thereby generating a signal with high spatial resolution. These colours are suitable for applications that assess the physiologic state of a specific tissue. Conversely, red and other maximally reflective pigments promote superficial scattering, thereby increasing the spatial range of the sensor and photodetector power. Therefore, red-coloured probes are well suited for assessing tissue viability and clinical applications, requiring miniaturized designs with lower power components and small batteries. We have found that red silicone enclosures increase raw photodetector values by a magnitude of 6 and cardiac pulsation amplitude by a factor of 3 compared to traditional black enclosures. The lower absorbance of red silicone, and the corresponding increase in sensor crosstalk, can be mitigated through the addition of TiO_2_ or other scattering agents. 

## Figures and Tables

**Table 1:** Dye and TiO_2_ concentrations for optical phantom preparation.

Dye Concentration	TiO_2_ Concentration		
	None	Low	High
0.27% Red (PMS 186C)	0%	0.018%	0.036%
0.27% Blue (PMS 2757C)	0%	0.018%	0.036%
0.27% Black (PMS Black)	0%	0.018%	0.036%

**Table 2:** Attenuation coefficients for prepared phantoms, averaged for *n* = 3 samples.

Colour	Attenuation Coefficient (mm^−1^)		
	0% TiO_2_	0.018% TiO_2_	0.036% TiO_2_
Red	0.102 ± 0.017	0.260 ± 0.011	0.444 ± 0.020
Blue	0.366 ± 0.009	0.542 ± 0.039	0.805 ± 0.016
Black	0.591 ± 0.011	0.737 ± 0.013	0.978 ± 0.040

**Table 3:** Raw photodetector values before and after application of black tape.

Probe	Raw Photodetector Values (mV)				
	950 nm	730 nm	810 nm	850 nm	650 nm
Red	3800	3800	3800	3800	3800
Black-taped	600	550	500	500	450

**Table 4:** Measured cardiac pulsation amplitude and peak width for baseline measurements ± 1 standard deviation.

Participant	Condition	Cardiac Pulsation Amplitude (ab)			Cardiac Pulsation Width (s)		
		Trial 1	Trial 2	Trial 3	Trial 1	Trial 2	Trial 3
P1	Red Probe	6.43 ± 0.55	13.29 ± 1.23	10.16 ± 1.15	0.74 ± 0.10	0.77 ± 0.13	0.68 ± 0.09
	Black-Taped	2.29 ± 0.64	8.42 ± 1.17	1.94 ± 0.51	0.71 ± 0.18	0.81 ± 0.08	0.60 ± 0.18
P2	Red Probe	6.64 ± 0.38	5.17 ± 0.45	3.50 ± 0.56	0.82 ± 0.04	0.75 ± 0.04	0.79 ± 0.05
	Black-Taped	2.64 ± 0.48	3.27 ± 0.91	2.26 ± 0.27	0.74 ± 0.14	0.70 ± 0.06	0.71 ± 0.17

**Table 5:** Calibrated LED currents for red and blue probes following wavelengths’ power adjustments for *n* = 1 animal experiments.

Probe	LED Current (mA)					
	730 nm	680 nm	760 nm	850 nm	910 nm	Total
Red	9.4	20.4	9.4	9.4	17.3	65.9
Blue	51.0	60.0	31.0	8.0	8.0	158

## Data Availability

The data presented in this study could be made available upon request to the corresponding author.
